# White matter tract differences in persistent post-traumatic headache, migraine, and healthy controls: a diffusion tensor imaging study

**DOI:** 10.1186/s10194-025-02084-2

**Published:** 2025-07-04

**Authors:** Rune Häckert Christensen, Haidar Muhsen Al-Khazali, Cédric Gollion, Basit Ali Chaudhry, Messoud Ashina, Håkan Ashina

**Affiliations:** 1 Department of Neurology, Danish Headache Center, Copenhagen University Hospital– Rigshospitalet, Copenhagen, Denmark; 2 Department of Clinical Medicine, Faculty of Health and Medical Sciences, University of Copenhagen, Copenhagen, Denmark; 3 Translational Research Center, Copenhagen University Hospital– Rigshospitalet, Copenhagen, Denmark; 4 Department of Neurology, University Hospital of Toulouse, Toulouse, France

## Abstract

**Background:**

Persistent post-traumatic headache (PPTH) frequently resembles migraine but may involve distinct white matter perturbations. We compared white matter fiber tracts among adults with PPTH, migraine, and healthy controls (HCs).

**Methods:**

This cross-sectional diffusion tensor imaging (DTI) study enrolled adults with PPTH, migraine, and HCs, who underwent a single 3T MRI session. Tract-based spatial statistics quantified white matter integrity. Outcome measures included fractional anisotropy (FA), axial diffusivity (AD), mean diffusivity, and radial diffusivity. Group comparisons used general linear models with threshold-free cluster enhancement and 5,000 permutations. Voxelwise significance was set at *P* < 0.05 after family-wise error correction, adjusting for age and sex. Post-hoc region-of-interest (ROI) analyses explored differences within significant regions.

**Results:**

Imaging data were available from 100 participants with PPTH, 293 with migraine, and 154 HCs. Compared to participants with migraine, those with PPTH exhibited higher FA and AD values in the posterior limb of the internal capsule and superior corona radiata (*P*_*FWE*_ < 0.05), findings that were further confirmed by post-hoc ROI analysis relative to HCs (both *P* < 0.001). FA values within these tracts were positively associated with monthly migraine-like headache days (β = 0.00045; *P* = 0.043). In addition, participants with PPTH showed lower AD values within the corpus callosum compared to those with migraine (*P*_*FWE*_ < 0.05), with post-hoc ROI analyses also demonstrating similar differences relative to HCs (*P* = 0.002). Lower corpus callosal AD values were associated with greater post-concussive symptom severity (β = − 0.0000013; *P* = 0.036). Mean diffusivity and radial diffusivity did not differ among groups.

**Conclusions:**

PPTH is associated with distinct white matter alterations involving ascending somatosensory pathways and interhemispheric fibers. These alterations are associated with clinical symptom severity and differentiate adults with PPTH from those with migraine, suggesting trauma-induced maladaptive plasticity.

**Supplementary Information:**

The online version contains supplementary material available at 10.1186/s10194-025-02084-2.

## Introduction

Persistent post-traumatic headache (PPTH) is a prevalent sequela of mild traumatic brain injury (mTBI) and presents significant challenges to understanding its neurobiological basis [1, 2]. Although participants with PPTH often display clinical features resembling migraine [3–5], the underlying mechanisms of PPTH remain poorly defined [1]. In contrast to migraine, which typically arises spontaneously and involves a genetic predisposition [6, 7], PPTH develops after head trauma and may reflect structural brain changes associated with mTBI [1, 8]. Clarifying the neurobiological distinctions between PPTH and migraine is critical for advancing knowledge of headache pathophysiology.

Diffusion tensor imaging (DTI) is a non-invasive technique that assesses white matter microstructure and has been widely applied in mTBI research [9, 10]. Prior DTI studies have documented widespread alterations in white matter integrity following mTBI [11–13]. However, relatively few investigations have specifically examined white matter changes in adults with PPTH [14–16], and direct comparisons among participants with PPTH, migraine, and healthy controls (HCs) remain limited. Moreover, previous research has been constrained by small sample sizes, heterogeneous methodologies, and inconsistent participant characterization [14–16].

To address these gaps, we conducted a large-scale analysis of white matter integrity in adults with PPTH, migraine, and HCs using tract-based spatial statistics (TBSS) applied to DTI metrics [17]. We hypothesized that participants with PPTH would exhibit distinct patterns of white matter alteration compared to participants with migraine and HCs. Specifically, we anticipated changes in fiber tracts implicated in nociceptive processing and structurally vulnerable to trauma. In addition, we explored whether the degree of white matter disruption correlated with headache burden and symptom severity, aiming to provide mechanistic insight into PPTH pathophysiology.

## Methods

The data presented are from the parental *Re*gistry *for M*igraine (REFORM), a prospective, single-center study [18, 19]. Participants with PPTH were enrolled as part of an extension to the REFORM study, following the same protocol and ethical approvals [20, 21]. The study adhered to the Declaration of Helsinki and received approval from the relevant ethics committee (Identifier: H-20033264). All participants provided written informed consent before study procedures commenced. The protocol was prospectively registered with ClinicalTrials.gov (Identifier: NCT04674020).

### Design and participants

This cross-sectional DTI study enrolled adults aged 18 years or older across three groups: participants with PPTH, participants with migraine, and HCs. Enrollment spanned from November 2020 to October 2023. Participants with PPTH and migraine were primarily recruited from the outpatient clinic of a national referral hospital. HCs were identified through an online advertisement posted on a Danish research participant website (https://forsoegsperson.dk/). Complete inclusion and exclusion criteria for all three groups are provided in Supplemental Tables 1–3.

Participants with PPTH had to meet the diagnostic criteria for persistent headache attributed to mild traumatic injury to the head in accordance with the International Classification of Headache Disorders, 3rd edition (ICHD-3) [2]. They also reported at least four headache days per month in the three months prior to enrollment. Main exclusion criteria included moderate or severe TBI, multiple TBIs, whiplash injury, neurologic disease, severe somatic illness, or any pre-mTBI primary headache disorder other than infrequent episodic tension-type headache.

Participants with migraine fulfilled ICHD-3 criteria for migraine without aura, with aura, or chronic migraine, with diagnosis established at least one year before enrollment. They also reported at least four migraine days per month during the preceding three months. Key exclusion criteria included comorbid neurologic conditions or severe somatic illness.

HCs were excluded if they had a history of any headache disorder other than infrequent episodic tension-type headache, a first-degree relative with a primary headache disorder, regular medication use, or any significant medical condition.

### Clinical procedures

At the study visit, participants completed a detailed semi-structured interview, recording headache characteristics and associated symptoms. For participants with ongoing headache at the time of MRI, features of the active headache were registered.

To minimize confounding factors, participants were instructed to abstain from analgesics, acute headache medications, anti-inflammatory drugs, sedatives, and antihistamines for 48 h before MRI scanning. All participants were also instructed to refrain from caffeinated beverages or foods for 12 h before the scan. Compliance was confirmed on the day of scanning.

Participants with PPTH and migraine completed the Headache Impact Test-6 (HIT-6) [22], Migraine Disability Assessment Scale (MIDAS) [23], and the Allodynia Symptoms Checklist-12 (ASC-12) [24]. Participants with PPTH also completed the Rivermead Post-Concussion Symptoms Questionnaire (RPQ) [25] and the Post-Traumatic Stress Disorder Checklist (PCL) [26].

### MRI procedures

MRI was performed on a 3.0 Tesla Siemens MAGNETOM Prisma scanner (Siemens Healthineers, Erlangen, Germany) with a 32-channel head coil. Participants were positioned with foam padding to minimize head motion artifacts and received instructions to remain as still as possible throughout the scan.

The imaging protocol included high-resolution structural imaging with a magnetization-prepared rapid gradient echo (MPRAGE) sequence, fluid-attenuated inversion recovery (FLAIR) imaging, and diffusion tensor imaging (DTI). DTI parameters were as follows: b = 1000 s/mm² and b = 0 s/mm²; repetition time (TR) = 11,000 ms; echo time (TE) = 60 ms; flip angle = 90°; field of view (FOV) = 256 × 256 mm²; 70 axial slices; slice thickness = 2 mm; and voxel size = 2 × 2 × 2 mm³; acquisition time = 6 min and 27 s. The full scan duration was about 55 min.

T1-weighted and FLAIR images were reviewed by a board-certified neuroradiologist blinded to participant group. Incidental findings were documented and participants with clinically significant abnormalities were excluded.

### Preprocessing

DTI data were preprocessed using the FMRIB Software Library (FSL), version 6.0.5 (Analysis Group, FMRIB, Oxford, United Kingdom). Images were corrected for motion and eddy current distortions using linear registration of the b = 0 volumes. Brain extraction followed, and all registrations and corrections were visually inspected to ensure accuracy. Diffusion tensors were then fitted to generate maps of fractional anisotropy (FA), axial diffusivity (AD), mean diffusivity, and radial diffusivity.

The TBSS pipeline was applied, including non-linear registration of all images to the 1 × 1 × 1 mm FMRIB58 template in MNI152 space using FNIRT. Mean FA images were skeletonized based on peak FA values, applying a threshold of ≥ 0.2 to identify central white matter tracts. Each participant’s FA map was projected onto the mean FA skeleton for voxelwise comparison. AD, mean diffusivity, and radial diffusivity maps were projected similarly for group-level analysis.

### Outcome measures

The imaging outcome measures included: FA, AD, mean diffusivity, and radial diffusivity. These DTI measures were selected based on their sensitivity to microstructural changes in white matter integrity [27, 28]. FA reflects the degree of directional diffusion of water molecules within axonal fibers [27, 28]. Higher FA values suggest greater fiber coherence, axonal density, or myelination, whereas lower FA values indicate potential axonal injury, demyelination, or loss of microstructural organization. Axial diffusivity quantifies diffusion parallel to the principal fiber direction [27, 28]. Higher AD values reflect adaptive reorganization or changes in axonal packing, whereas lower AD values suggest axonal injury or cytoskeletal disruption. Mean diffusivity measures the average magnitude of diffusion regardless of direction, providing a general marker of tissue density and structural integrity [27, 28]. Radial diffusivity assesses diffusion perpendicular to the principal axis and is commonly associated with myelin integrity [27, 28]. The clinical outcome measures included monthly headache days, monthly migraine-like headache days, HIT-6 scores, MIDAS scores, ASC-12 scores, RPQ scores, and PCL scores.

### Comparison groups

We compared outcome measures between participants with PPTH and HCs, and between participants with PPTH and those with migraine. Detailed comparisons between participants with migraine and HCs are previously reported [29], and were not the focus of the current study. For post-hoc region-of-interest (ROI) analyses, mean DTI measures were extracted from clusters showing significant group differences. These DTI measures were then tested for associations with clinical variables in the PPTH group, including monthly headache days, disease duration, and scores from the HIT-6, MIDAS, RPQ, PCL, and ASC-12.

### Statistical analysis

Demographic and clinical characteristics were summarized using means and standard deviations or medians and interquartile ranges, depending on data distribution. Normality of continuous variables was assessed through visual inspection of histograms and Q–Q plots. Homoscedasticity was evaluated with Levene’s test. The distribution of continuous demographic variables, such as age, was further compared using the two-sample Kolmogorov-Smirnov test. Comparisons of continuous variables between groups were performed using Student’s unpaired t-tests for normally distributed data and Wilcoxon rank-sum tests for skewed data. Categorical variables were compared using Chi-square or Fisher’s exact tests, as appropriate. All statistical analyses of clinical data were performed in R (version 4.2.0), with significance set at *P*  0.05.

DTI data were analyzed using FSL with the TBSS package [17]. All data were preprocessed and analyzed on a workstation running macOS Big Sur (version 11.7.10). Group comparisons used general linear models (GLMs) with threshold-free cluster enhancement (TFCE), 5000 permutations, and voxel-wise significance set at *P* < 0.05 after familywise error correction, adjusting for age and sex [30]. This approach avoids arbitrary cluster thresholds and improves sensitivity while maintaining low false positive rates [30].

Specific voxel-level *P*-values from TFCE analyses are not individually reported, in accordance with TFCE standards, to prevent misleading interpretations. Instead, the applied thresholds and results are detailed in the text and figures. Significant results were labeled according to the Johns Hopkins University (JHU) International Consortium for Brain Mapping (ICBM) DTI-81 White-Matter Labels Atlas [31]. For display purposes, significant voxels were filled using tbss_fill.

For post-hoc ROI analyses, we extracted mean DTI values from significant voxels to examine associations with clinical variables in participants with PPTH. These variables included monthly migraine-like days, monthly headache days, disease duration, average headache intensity (calculated as the most typical headache intensity of the most frequent headache type), and scores from the HIT-6, MIDAS, RPQ, PCL, and ASC-12. Associations were tested using generalized linear models adjusted for age and sex, with significance set at *P* < 0.05. To validate model assumptions, parallel analyses were conducted using generalized additive models, which produced statistically similar results.

We conducted additional post-hoc ROI analyses by extracting averaged DTI measures within regions significantly different in whole-brain analysis. These were further examined within GLMs. To control for confounding factors, a multivariate model was used adjusting for age, sex, use of preventive medication, depression, anxiety, presence of headache at scan, and frequency of acute medication use.

We also performed subgroup comparisons within these ROIs, comparing participants with PPTH to different migraine subtypes, including migraine without aura, migraine with aura, chronic migraine, and episodic migraine. These comparisons were analyzed using both models adjusted for age and sex, as well as a more comprehensive multivariate model using the same confounders listed above. Finally, additional subgroup ROI analyses were conducted restricted to female participants adjusted for age. For all post-hoc analyses, significance was set at *P* ≤ 0.05.

## Results

Between November 2020 and October 2023, 105 participants with PPTH, 306 with migraine, and 160 HCs completed a 3 T MRI scan. After excluding individuals with incidental findings or poor scan quality, the final sample included 100 participants with PPTH, 293 with migraine, and 154 HCs (Table 1).

**Fig. 1:**
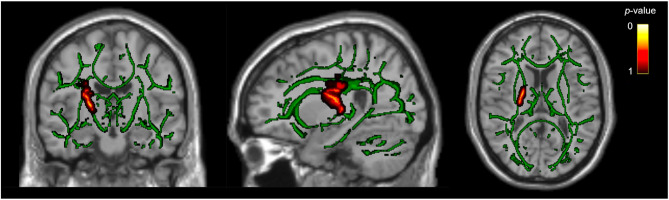
Greater Fractional Anisotropy in Right-Sided White Matter Regions in PPTH Compared to Migraine. Voxels with significantly higher fractional anisotropy (FA) in participants with persistent post-traumatic headache compared to those with migraine are shown in hot colors. Images in radiological convention. These voxels are located in the right posterior limb of the internal capsule and the superior corona radiata. Results are overlaid on the FA skeleton (green) for reference. Statistical threshold *P*_FWE_ < 0.05; number of voxels = 508; MNI coordinates: X: 23, Y: -12, Z: 20; filled for display purposes

**Table 1:** Sociodemographic and Clinical Characteristics of the Study Populations

Characteristics	PPTH	Healthy Controls	Migraine	*P*-value (PPTH vs Migraine)	*P*-value (PPTH vs HC)
No. of Participants	100	154	293	-	-
Sex (Male:Female)	25:75	21:133	34:259	0.001*	0.022
Age, Mean (SD) [Range]	40.8 (11.7)	41.5 (11.6)	41.6 (12.3)	0.573	0.606
Right-Handed, n (%)	88 (88.0)	133 (86.4)	271 (92.4)	0.168	0.705
Migraine With Aura, n (%)	NA	NA	103 (35.2)	-	-
Chronic Migraine, n (%)	NA	NA	181 (61.8)	-	-
Monthly Migraine(-Like) Days, Mean (SD)	14.0 (12.0)	NA	13.3 (6.8)	0.399	-
Monthly Headache Days, Mean (SD)	25.2 (9.3)	NA	18.9 (8.1)	< 0.001*	-
Monthly Number of Days with Acute Medication Use, Mean (SD)	8.9 (8.6)	NA	11.2 (6.4)	< 0.001*	-
Current Preventive Medication Use, n (%)†	37 (37.0)	NA	165 (56.3)	< 0.001*	-
Medication-Overuse Headache, n (%)	12 (12.0)	NA	108 (36.8)	< 0.001*	-
Disease Duration, Years, Mean (SD)	8.6 (6.0)	NA	22.8 (12.3)	< 0.001*	-
Comorbid Depression, n (%)	34 (34.0)	NA	37 (12.7)	< 0.001*	-
Comorbid Anxiety, n (%)	23 (23.0)	NA	31 (10.6)	0.002	-
Headache During Scan, n (%)					
Migraine(-Like) Headache††	6 (6.0)	NA	79 (26.9)	< 0.001*	-
Non-Migraine Headache†††	85 (85.0)	NA	124 (42.3)	< 0.001*	-
Headache-Free	9 (9.0)	NA	90 (30.7)	< 0.001*	-

*Abbreviations*: *HC* healthy controls, *PPTH* persistent post-traumatic headache, *SD* standard deviation

*Significant at *P *≤ 0.05

†Including riboflavin and magnesium

††Meets ICHD-3 criteria for migraine without aura (code 1.1) and is of at least moderate to severe intensity (Numerical Rating Scale 4–10)

†††Headache that does not meet ICHD-3 criteria for migraine without aura (code 1.1)

Participants with PPTH had a mean age of 40.8 years (SD, 11.7), and 75 (75%) were female. They reported a mean of 25.2 headache days per month (SD, 9.3) and used acute headache medications on 8.9 days per month (SD, 8.6). Thirty-seven (37%) were using preventive migraine medications at enrollment (See Supplemental Table 4 for types of preventives in PPTH and migraine). During MRI acquisition, 91 (91%) participants with PPTH reported experiencing an active headache.

The PPTH and migraine groups were well balanced in terms of age (*P* = 0.57) and age distribution (*P* = 0.70). No significant differences were observed between participants with PPTH and HCs in age (*P* = 0.61) or age distribution (*P* = 0.90). However, a higher proportion of males was observed among participants with PPTH compared with participants with migraine (*P* = 0.001) and HCs (*P* = 0.022).

### Fractional anisotropy

Whole-brain analysis showed that participants with PPTH had significantly higher FA values in the right posterior limb of the internal capsule and superior corona radiata compared with participants with migraine (*P*_*FWE*_ < 0.05). The significant regions included 508 voxels, with peak MNI coordinates at X = 23, Y = − 12, Z = 20 (Table 2; Fig. 1; Supplemental Fig. 1).

**Table 2:** Overview of significant Whole-brain analysis results

Comparison	DTI Measure	Predominant Region	Peak Voxel t-Score	Volume of Significant Cluster (k)	MNI Coordinates (Peak Voxel)			MNI Coordinates (Center of Gravity)		
					X	Y	Z	X	Y	Z
PPTH vs. Migraine	↑FA in PTH	Right posterior limb of internal capsule & superior corona radiata	5.34	479	23	−12	20	24	−11	15
			4.33	26	26	−19	26	26	−19	27
			3.34	3	27	−12	29	28	−11	29
	↑AD in PTH	Left posterior limb of internal capsule & superior corona radiata	4.49	163	−23	−10	16	−24	−9	15
			4.49	44	−24	−15	34	−24	−15	34
			4.5	1	−27	−11	28	−27	−11	28
	↓AD in PTH	Corpus callosum	5.12	141	−6	−24	25	−4	−27	24
PPTH vs. HC	All	No significant differences	-	-	-	-	-	-	-	-
Migraine vs. HC	All	No significant differences	-	-	-	-	-	-	-	-

*Abbreviations*: *AD* axial diffusivity, *DTI* diffusion tensor imaging, *FA* fractional anisotropy, *HC* healthy controls, *MNI* Montreal Neurological Institute, *PPTH* persistent post-traumatic headache

No significant whole-brain FA differences were observed between participants with PPTH and HCs. However, post-hoc ROI analysis, restricted to the identified regions, showed higher FA values in participants with PPTH compared with HCs (*P* = 0.002; Supplemental Table 5).

Among participants with PPTH, higher FA values within the identified regions were associated with a greater number of monthly migraine-like headache days (β = 0.00045; 95% CI, 0.00002–0.0089; *P* = 0.043. See Table 3).

**Table 3:** Associations between clinical variables and significant DTI metrics

Clinical variable	FA values in significant regions of internal capsule and corona radiata	AD values in significant regions of internal capsule and corona radiata	AD values in significant region in corpus callosum
Monthly migraine-like days	0.043*	0.436	0.800
MHD	0.820	0.710	0.689
HIT-6 score	0.681	0.507	0.617
MIDAS score	0.930	0.473	0.103
RPQ score	0.712	0.203	0.036*
PCL grade	0.631	0.122	0.113
ASC-12 score	0.308	0.635	0.533
Disease duration	0.948	0.247	0.101
Average headache intensity	0.136	0.983	0.218

*Abbreviations:* *AD* axial diffusivity, *ASC-12* 12-item Allodynia Symptom Checklist, *FA* fractional anisotropy, *HIT-6* Headache Impact Test, *MHD* monthly headache days, *MIDAS* Migraine disability Assessment Test, *PCL* PTSD checklist for DSM-5, *RPQ* Rivermead Post Concussion Symptoms Questionnaire

* Significant at *P* ≤ 0.05

### Axial diffusivity

**Fig. 2:**
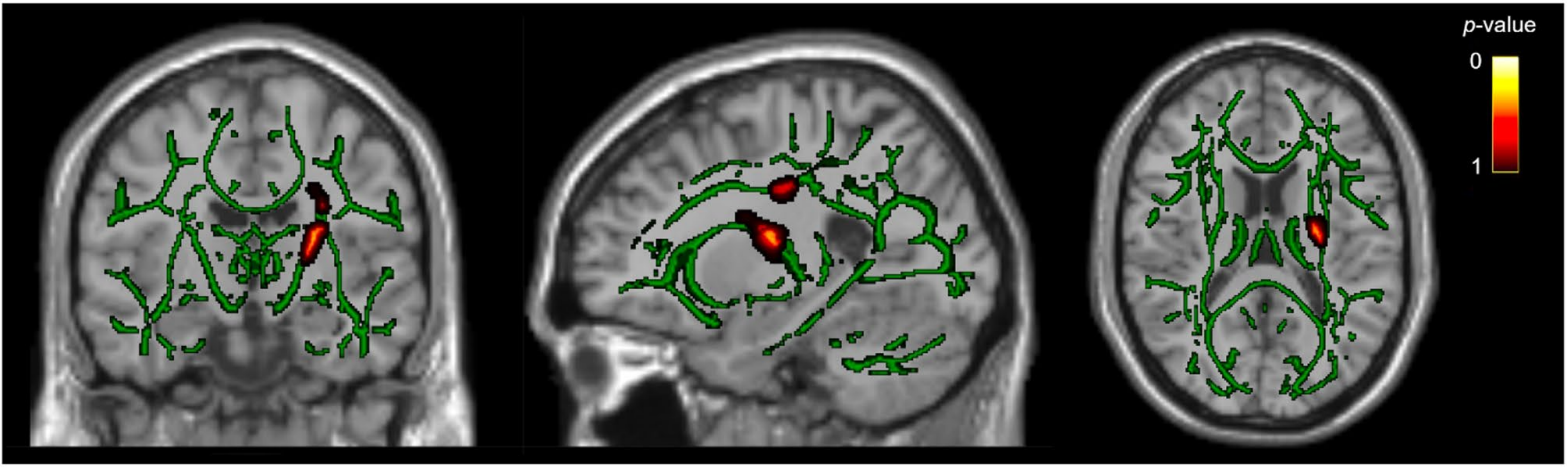
Greater Axial Diffusivity in Left-Sided White Matter Regions in PPTH Compared to Migraine. Voxels with significantly higher axial diffusivity (AD) in PPTH compared to migraine are shown in hot colors. Images in radiological convention. The significant regions include the left posterior limb of the internal capsule and the superior corona radiata. Voxels are overlaid on the FA skeleton (green). Statistical threshold: *P*_*FWE*_ < 0.05; number of voxels = 208; MNI coordinates: X = − 23, Y = − 10, Z = 16; filled for display purposes

Whole-brain analysis demonstrated that participants with PPTH had higher AD values in the left posterior limb of the internal capsule and superior corona radiata compared with participants with migraine (*P*_*FWE*_ < 0.05). The significant regions comprised 208 voxels, with peak MNI coordinates at X = − 23, Y = − 15, Z = 34 (Table 2; Fig. 2; Supplemental Fig. 1).

No significant whole-brain AD differences were observed between participants with PPTH and HCs. However, post-hoc ROI analysis, restricted to the identified regions, revealed that AD values were higher in participants with PPTH compared with HCs (*P* < 0.001).

**Fig. 3:**
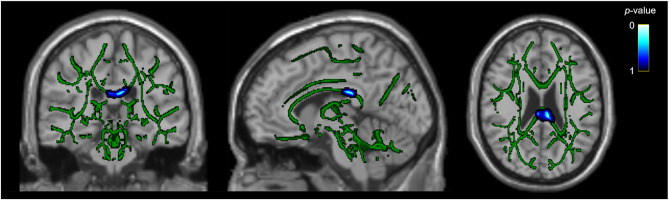
Lower Axial Diffusivity in the Corpus Callosum in PPTH Compared to Migraine.Voxels with significantly lower axial diffusivity (AD) in participants with PPTH compared to those with migraine are shown in cold colors. Images in radiological convention. The regions include the corpus callosum. Data are superimposed on the FA skeleton (green) for reference. Statistical threshold: *P*_*FWE*_ < 0.05; number of voxels = 141; MNI coordinates: X = − 6, Y = − 24, Z = 25; filled for display purposes

Whole-brain analysis also identified lower AD values in participants with PPTH compared with those with migraine within the corpus callosum (*P*_*FWE*_ < 0.05). This finding encompassed 141 significant voxels, with peak MNI coordinates at X = − 6, Y = − 24, Z = 25 (Fig. 3; Supplemental Fig. 1; Supplemental Table 5). Post-hoc ROI analysis showed that AD values in this corpus callosum region were lower in participants with PPTH, compared with HCs (*P* = 0.002; Supplemental Table 5). Among participants with PPTH, lower AD values were associated with higher RPQ-scores (β = − 0.0000013; 95% CI, − 0.0000025 to − 0.00000011; *P* = 0.036. See Table 3).

### Mean diffusivity and radial diffusivity

No significant differences in mean diffusivity or radial diffusivity were observed between groups in the whole-brain analyses.

### Post-hoc ROI analyses

Multivariate post-hoc ROI analyses were performed in all three regions that showed significant between-group differences in DTI-derived metrics (Table 2; Figs. 1, 2 and 3). The observed group differences remained statistically significant even after adjusting for age, sex, preventive medication use, comorbid depression, comorbid anxiety, number of days per month with acute medication use, and headache at the time of scanning (Supplemental Tables 6–8). Furthermore, age was consistently and negatively associated with both FA and AD values across all three regions. In the corpus callosum, preventive medication use was also significantly associated with lower AD values (*P* < 0.011), but it was not associated with DTI-derived metrics in the other regions. No significant associations were found for comorbid depression, comorbid anxiety, active headache during scan, or number of days per month with acute medication use in any model.

Further post-hoc analyses revealed that the primary findings remained significant across all migraine subtypes (i.e., migraine with aura, migraine without aura, episodic migraine, chronic migraine) in multivariate models (all *P* < 0.05; Supplemental Table 9).

In post-hoc analyses restricted to female participants with PPTH (*n* = 75), the findings remained significant in all three regions compared with HCs (*P* < 0.01; Supplemental Table 10).

## Discussion

This cross-sectional DTI study identified distinct white matter microstructural alterations in adults with PPTH compared to those with migraine and HCs. Participants with PPTH exhibited higher FA and AD values in the posterior limb of the internal capsule and superior corona radiata. They also showed lower AD values within the corpus callosum. These alterations were associated with greater clinical symptom severity, suggesting trauma-induced maladaptive plasticity within ascending somatosensory pathways and disrupted interhemispheric connectivity. Together, our findings differentiate PPTH from migraine at a structural level and highlight potential imaging biomarkers to improve disease characterization.

### Alterations in ascending somatosensory pathways

Higher FA and AD values within the posterior limb of the internal capsule and superior corona radiata suggest microstructural remodeling of somatosensory tracts involved in nociceptive transmission. Although standard DTI atlases do not separate nociceptive from motor fibers [32], these findings align with the known anatomical trajectory of thalamocortical pain projections [33–35].

Within the trigeminovascular system—the primary substrate for persistent post-traumatic headache and migraine—nociceptive inputs from cranial structures ascend through the trigeminal nerve, brainstem nuclei, and thalamic nuclei [36]. From the thalamus, these signals project to the primary somatosensory cortex via fibers coursing through the posterior limb of the internal capsule and superior corona radiata [33–36]. In the posterior limb of the internal capsule, these fibers are tightly bundled before fanning into the corona radiata en route to the postcentral gyrus [33–35]. Structural alterations in the posterior limb of the internal capsule and superior corona radiata therefore implicate disruption or plasticity along the final relay of trigeminovascular nociceptive processing toward cortical networks.

Increased FA and AD values might reflect neuroplastic adaptations, including enhanced axonal packing, increased myelination, or reorganized fiber coherence in response to persistent nociceptive drive [37–41]. Similar plastic changes have been observed after motor learning, sensory enrichment, and sustained peripheral stimulation in adults [37–41]. In PPTH, chronic nociceptive input—potentially driven by meningeal injury, neurogenic inflammation, or dural sensitization—could promote long-term remodeling of ascending pathways. Somewhat supporting this hypothesis, FA values were weakly but positively associated with monthly migraine-like headache frequency in PPTH. That this relationship was not observed in migraine suggests potential trauma-specific remodeling, though this interpretation remains speculative and requires further validation. This interpretation aligns with prior findings, in which we observed altered cortical morphometry within the postcentral gyrus of participants with PPTH [21]. These changes were characterized by an enlarged cortical surface area, which might reflect plastic alterations associated with intensive sensory processing [21].

Notably, no comparable microstructural alterations were observed in participants with migraine (see [29] for previous report). This difference might reflect fundamental distinctions in pathophysiology. PPTH often presents with continuous or near-daily headache [4], suggesting a more severe or sustained nociceptive burden. In contrast, migraine often involves a genetic predisposition or possibly altered central excitability, generating episodic pain without necessarily inducing structural changes in ascending tracts [6, 7].

### Disruptions in corpus callosum

The corpus callosum connects the cerebral hemispheres and synchronizes interhemispheric functions [42–44]. Due to its central location and dense fiber concentration, it is particularly vulnerable to shear strain during trauma [45]. Our results showed that participants with PPTH exhibited lower AD values in the corpus callosum compared to those with migraine and HCs. These findings were localized to the isthmus of the corpus callosum, which contains fibers linking the somatosensory and primary auditory cortices [46]. Lower AD values in this region might reflect altered sensory integration, potentially contributing to symptoms such as headache and phonophobia in participants with PPTH. Supporting this, AD values in the isthmus were negatively associated with RPQ scores among participants with PPTH. It is worth noting that the RPQ captures a broad range of symptoms following TBI, including headache, phonophobia, cognitive dysfunction, and fatigue [25]. Thus, structural alterations in the corpus callosum might reflect broader sensory and cognitive symptomatology in PPTH, compared with migraine.

Previous DTI studies have implicated disruption of the corpus callosum following mTBI [47]. Early investigations demonstrated persistent reductions in FA across the genu, body, and splenium, suggesting chronic axonal injury and demyelination [47]. Kim et al. reported neurite density changes in the genu associated with post-concussive symptoms [9]. Longitudinal studies have also found progressive reductions in FA within the midbody and splenium after mTBI [48]. Specific to PPTH, Chong et al. reported reduced FA in the posterior corpus callosum, which was associated with symptom severity and reduced quality of life [14]. Together, these findings implicate the corpus callosum as a critical structure involved in both the immediate and long-term consequences of mTBI.

### Existing evidence and added value

Few studies have specifically investigated DTI alterations in PPTH [14–16], despite the extensive DTI literature in mTBI without headache pattern characterization. Existing studies generally report widespread white matter changes suggestive of reduced integrity, although findings have been inconsistent [49–51]. Our results do not support widespread injury but instead identifies localized alterations in pain-related tracts, some of which might reflect plasticity rather than degeneration [52].

Several factors likely contributed to the specificity of our findings. First, we enrolled a larger sample size (*n* = 547) compared to previous studies (ranging from 24 to 131 participants) [14–16]. Second, we used a conservative voxel-wise statistical approach with validated TFCE [30]. Third, we applied TBSS, which reduces intersubject variability compared to tractographic methods [17]. Fourth, differences in sample characteristics likely influenced white matter findings. Mechanisms of TBI vary geographically, with blast-related concussions and sports injuries more common in U.S. cohorts [14, 15] and falls or unintentional injuries more frequent in European populations [4, 20]. These mechanisms might induce different neurobiological consequences, despite a shared ICHD diagnosis. Supporting this, machine learning classifiers using DTI features have more accurately distinguished PPTH after blast injury than after sports trauma or falls [15].

Future research should determine whether early DTI alterations predict headache persistence and symptom burden. Longitudinal imaging from acute to persistent phases will be essential to differentiate maladaptive plasticity from primary trauma effects. Although TBSS addresses several limitations of tractography, complementary approaches such as tractography and mapping the structural connectome could provide additional insight into white matter changes in PPTH.

### Strengths and limitations

The strengths of this study include its large sample size, use of a high-resolution 3 T MRI scanner, and conservative neuroimaging statistics. The inclusion of both a migraine comparator group and HCs allows a nuanced understanding of PPTH-specific changes relative to primary headache and non-headache populations. The combination of whole-brain and region-of-interest (ROI) analyses strengthens the robustness of our findings. However, several limitations should be acknowledged. The cross-sectional design limits causal inference about the temporal relationship between injury, microstructural alterations, and clinical symptoms. Our sample was predominantly female, mirroring headache epidemiology but potentially limiting generalizability. While diffusion metrics are sensitive to microstructural changes, they are non-specific and cannot definitively distinguish mechanisms such as demyelination, axonal injury, or inflammation without additional imaging modalities. Although not all comparisons were fully age- and sex-matched, all analyses statistically controlled for these covariates. Furthermore, post-hoc analyses demonstrated that the primary findings remained robust after adjustment for a broad range of clinical and demographic covariates. The observed alterations were consistent across migraine subtypes, supporting the specificity of PPTH-related changes. Lastly, we did not investigate individuals with acute PTH. Future longitudinal studies are needed to examine whether the observed changes originate acutely after trauma or develop as secondary plastic adaptations.

## Conclusions

This DTI investigation identifies distinct and localized white matter changes in adults with PPTH compared to those with migraine and HCs. Alterations were observed within ascending somatosensory pathways and interhemispheric fiber tracts. These findings advance our understanding of the neurobiological mechanisms underlying persistent headache post-mTBI. Future longitudinal and multimodal imaging studies are needed to map the temporal progression of these changes and to possibly inform efforts for early intervention to prevent chronic headache development.

## Supplementary Information

Supplementary Material 1.

## Data Availability

Data will be made available upon reasonable request from a qualified investigator.

## Abbreviations

AD: Axial diffusivity
DTI: Diffusion tensor imaging
FA: Fractional anisotropy
FLAIR: Fluid-attenuated inversion recovery
FMRIB: Functional Magnetic Resonance Imaging of the Brain (FMRIB) 58-subject average template
FSL: FMRIB Software Library
MIDAS: Migraine Disability Assessment Scale
MNI: Montreal Neurological Institute
MRI: Magnetic resonance imaging
PPTH: Persistent post-traumatic headache
PTH: Post-traumatic headache
ROI: Region of interest
RPQ: Rivermead Post Concussion Symptoms Questionnaire
TBI: Traumatic brain injury
TBSS: Tract-based spatial statistics
TFCE: Threshold-free cluster enhancement
