# Supplementary material for: White matter tract differences in persistent post-traumatic headache, migraine, and healthy controls: a diffusion tensor imaging study
Source: J Headache Pain. 2025 Jul 4;26(1):155. doi: 10.1186/s10194-025-02084-2 (PMC12226909; doi:10.1186/s10194-025-02084-2)
Supplement: Supplementary file 1 — Supplementary Material 1. [file 10194_2025_2084_MOESM1_ESM.docx]

**Supplementary Materials**

**Supplemental Table 1. Inclusion and Exclusion Criteria for Participants with Persistent Post-Traumatic Headache**

| **Inclusion Criteria** | **Exclusion Criteria** |
| --- | --- |
| - Age ≥18 years at screening - Persistent headache attributed to mild traumatic brain injury for ≥ 12 months (ICHD-3, code 5.2.2) - ≥4 headache days per month on average over the 3 months prior to screening - Signed informed consent before any study-specific procedures | - History of more than one mild traumatic head injury - History of moderate or severe traumatic brain injury - History of whiplash injury - History of craniotomy - Any other clinically significant condition (except as listed) that might pose a risk to safety or interfere with study assessments - Inability to distinguish migraine from other headache types - History of suicidal behavior or risk of harm to self or others - Positive pregnancy test at any study visit (females of childbearing potential) - Current pregnancy or breastfeeding - Use of preventive medication other than anti-CGRP antibodies unless dose was stable for ≥ 2 months prior to baseline |

Modified from Christensen et al. [1].

**Supplemental Table 2. Inclusion and Exclusion Criteria for Participants with Migraine**

| **Inclusion Criteria** | **Exclusion Criteria** |
| --- | --- |
| - Age ≥18 years at screening - Diagnosis of migraine without aura, migraine with aura, or chronic migraine for ≥ 12 months per ICHD-3 criteria (codes 1.1, 1.2, or 1.3) - ≥4 migraine days per month on average during the 3 months prior to screening - Signed informed consent before any study-specific procedures - Scheduled preventive treatment with erenumab | - Migraine onset after age 50 - History of persistent post-traumatic headache, hemiplegic migraine, or cluster headache (ICHD-3 codes 5.2, 1.2.3, 3.1) - Inability to distinguish migraine from other headache types - History of suicidal behavior or risk of harm to self or others - Any other clinically significant condition (except as listed) that might pose a risk to safety or interfere with study assessments - Previous treatment with erenumab - Treatment with another anti-CGRP monoclonal antibody within 3 months prior to the first study visit - Use of other preventive medication (excluding anti-CGRP antibodies) unless dosage was stable for at least 2 months prior to baseline |

Modified from Christensen et al. [2].

**Supplemental Table 3. Inclusion and Exclusion Criteria for Healthy Controls**

| **Inclusion Criteria** | **Exclusion Criteria** |
| --- | --- |
| - Age ≥ 18 years at the screening visit | - History of primary headache disorders (except infrequent episodic tension-type headache) - History of secondary headache disorders - First-degree relative with a history of any primary headache disorder (except tension-type headache ≤ 5 days/month) - Headache occurring within 24 hours of any study-related procedure - History of suicidal behavior or risk of self-harm - Daily use of medications (excluding oral contraceptives) - Any clinically significant disorder, condition, or disease (at investigator's discretion) that might pose a risk or interfere with study participation or evaluation - History or evidence of any psychiatric disorder - Positive pregnancy test during the study visit (females of childbearing potential) - Current pregnancy or breastfeeding; unwillingness to use an acceptable method of effective contraception (e.g., oral contraceptives, IUD, barrier methods, abstinence) - Contraindications to MRI - Unlikely to comply with study procedures or complete participation (based on investigator judgment) |

Modified from Christensen et al. [2].

**Supplemental Table 4. Preventive Headache Treatment in Participants with PPTH and Migraine.**

| **Preventive Headache Treatment, n (%)** | **PPTH  (N = 100)** | **Migraine  (N = 293)** |
| --- | --- | --- |
| Metoprolol | 2 (2.0) | 25 (8.5) |
| Propranolol | 0 (0.0) | 6 (2.0) |
| Bisoprolol | 0 (0.0) | 1 (0.3) |
| Candesartan | 9 (9.0) | 60 (20.5) |
| Lisinopril | 0 (0.0) | 6 (2.0) |
| Amitriptylin | 6 (6.0) | 20 (6.8) |
| Topiramat | 3 (3.0) | 13 (4.4) |
| Valproate | 0 (0.0) | 1 (0.3) |
| Riboflavin | 0 (0.0) | 6 (2.0) |
| Magnesium | 4 (4.0) | 22 (7.5) |
| OnabotulinumtoxinA | 7 (7.0) | 54 (18.4) |
| Gabapentin | 3 (3.0) | 3 (1.0) |
| GON block | 1 (1.0) | 0 (0.0) |
| Mirtazapine | 3 (3.0) | 1 (0.3) |
| Nortriptyline | 3 (3.0) | 0 (0.0) |
| Other | 7 (7.0) | 1 (0.3) |
| **Use of Any Preventive Headache Treatment** | **37 (37.0)** | **165 (56.3)** |

Abbreviations: GON, greater occipital nerve; PPTH, persistent post-traumatic headache.

**Supplemental Table 5. Post-Hoc ROI Comparisons of Significant DTI Metrics Between PPTH and HCs Identified from Whole-Brain Analyses**

|  | Region of Interest | Effect estimate (regression coefficient, β) | Standard error | *T*-value | *P*-value |
| --- | --- | --- | --- | --- | --- |
| PPTH > Healthy Controls | FA of right internal capsule and corona radiata region | 0.0097064 | 0.0031574 | 3.074 | 0.0024** |
|  | AD of left internal capsule and corona radiata region | 0.0000196 | 0.0000056 | 3.483 | 0.0006*** |
|  | AD of corpus callosum region | -0.0000258 | 0.0000081 | -3.171 | 0.0017** |

Abbreviations: AD, axial diffusivity; DTI, diffusion tensor imaging; FA, fractional anisotropy; MRI, magnetic resonance imaging; PPTH, persistent post-traumatic headache; ROI, region of interest.

*Significant at *P* ≤ 0.05. **Significant at *P* ≤ 0.01. ***Significant at *P* ≤ 0.001.

**Supplemental Table 6. Multivariate Analysis of Clinical Variables in Relation to Fractional Anisotropy in Regions with Greater Values in Persistent PPTH than in Migraine.**

| Variable | Effect estimate (regression coefficient, β) | Standard error | *T*-value | *P*-value |
| --- | --- | --- | --- | --- |
| PPTH > migraine | 0.0120841 | 0.0031631 | 3.820 | 0.0002*** |
| Age | -0.0002077 | 0.0001043 | -1.991 | 0.0472* |
| Sex | -0.0042613 | 0.0035920 | -1.186 | 0.2362 |
| Depression | 0.0004852 | 0.0035684 | 0.136 | 0.8919 |
| Anxiety | -0.0043850 | 0.0039088 | -1.122 | 0.2627 |
| Headache during MRI scan | -0.0046969 | 0.0029682 | -1.582 | 0.1144 |
| Preventive Medication Use | -0.0025395 | 0.0025377 | -1.001 | 0.3176 |
| Number of Days per Month with Acute Medication Use | -0.0002102 | 0.0001810 | -1.161 | 0.2462 |

Abbreviations: PPTH, persistent post-traumatic headache; MRI, magnetic resonance imaging.

*Significant at *P* ≤ 0.05. **Significant at *P* ≤ 0.01. ***Significant at *P* ≤ 0.001.

**Supplemental Table 7. Multivariate Analysis of Clinical Variables in Relation to Axial Diffusivity in Regions with Greater Values in Persistent PPTH than in Migraine.**

| Variable | Effect estimate (regression coefficient, β) | Standard error | *T*-value | *P*-value |
| --- | --- | --- | --- | --- |
| PPTH > migraine | 0.0000241 | 0.0000055 | 4.407 | <0.0001*** |
| Age | -0.0000005 | 0.0000002 | -3.002 | 0.0029** |
| Sex | -0.0000058 | 0.0000062 | -0.930 | 0.3530 |
| Depression | 0.0000002 | 0.0000062 | 0.028 | 0.9780 |
| Anxiety | -0.0000020 | 0.0000068 | -0.289 | 0.7727 |
| Headache during MRI scan | -0.0000027 | 0.0000051 | -0.523 | 0.6014 |
| Preventive Medication Use | -0.0000053 | 0.0000044 | -1.213 | 0.2259 |
| Number of Days per Month with Acute Medication Use | -0.0000002 | 0.0000003 | -0.560 | 0.5755 |

Abbreviations: PPTH, persistent post-traumatic headache; MRI, magnetic resonance imaging.

*Significant at *P* ≤ 0.05. **Significant at *P* ≤ 0.01. ***Significant at *P* ≤ 0.001.

**Supplemental Table 8. Multivariate Analysis of Clinical Variables in Relation to Axial Diffusivity in Regions with Lower Values in Persistent PPTH than in Migraine.**

| Variable | Effect estimate (regression coefficient, β) | Standard error | *T*-value | *P*-value |
| --- | --- | --- | --- | --- |
| PPTH > migraine | -0.0000332 | 0.0000079 | -4.182 | <0.0001*** |
| Age | -0.0000013 | 0.0000003 | -4.938 | <0.0001*** |
| Sex | 0.0000292 | 0.0000090 | 3.237 | 0.0013** |
| Depression | -0.0000107 | 0.0000090 | -1.198 | 0.2316 |
| Anxiety | 0.0000116 | 0.0000098 | 1.181 | 0.2384 |
| Headache during MRI scan | -0.0000036 | 0.0000075 | -0.483 | 0.6294 |
| Preventive Medication Use | -0.0000162 | 0.0000064 | -2.549 | 0.0112* |
| Number of Days per Month with Acute Medication Use | -0.0000002 | 0.0000005 | -0.363 | 0.7172 |

Abbreviations: MRI, magnetic resonance imaging, PPTH, persistent post-traumatic headache.

*Significant at *P* ≤ 0.05. **Significant at *P* ≤ 0.01. ***Significant at *P* ≤ 0.001.

**Supplemental Table 9. Post-Hoc ROI Analysis of DTI-Derived Metrics in PPTH Compared with Migraine Subtypes.**

| Post-hoc regions of interest | PPTH (N = 100) vs | | | |
| --- | --- | --- | --- | --- |
|  | Migraine without aura (N = 190)  (*P*-value) | Migraine with aura (N = 103)  (*P*-value) | Chronic migraine (N = 181)  (*P*-value) | Episodic migraine (N = 112)  (*P*-value) |
| ***Model adjusted for age and sex*** | | | | |
| FA of right internal capsule and corona radiata region | < 0.0001*** | 0.0004*** | < 0.0001*** | 0.0005*** |
| AD of left internal capsule and corona radiata region | < 0.0001*** | < 0.0001*** | < 0.0001*** | < 0.0001*** |
| AD of corpus callosum region | < 0.0001*** | < 0.0001*** | < 0.0001*** | 0.0001*** |
| ***Model adjusted for age, sex, anxiety, depression, ongoing headache, preventive treatment, and frequency of acute medication use*** | | | | |
| FA of right internal capsule and corona radiata region | < 0.0001*** | 0.0002*** | 0.0001*** | 0.0001*** |
| AD of left internal capsule and corona radiata region | < 0.0001*** | 0.0002*** | < 0.0001*** | < 0.0001*** |
| AD of corpus callosum region | 0.0007*** | 0.0008*** | 0.0002*** | 0.0024** |

Abbreviations: AD, axial diffusivity; DTI, diffusion tensor imaging; FA, fractional anisotropy; PTH, persistent post-traumatic headache.

*Significant at *P* ≤ 0.05. **Significant at *P* ≤ 0.01. ***Significant at *P* ≤ 0.001.

**Supplemental Table 10. Post-hoc ROI Analysis of Associations with Persistent Post-Traumatic Headache in Females.**

| Post-hoc regions of interest | Female participants with PPTH (N = 75)  (*P*-value) |
| --- | --- |
| FA of right internal capsule and corona radiata region | 0.0012** |
| AD of left internal capsule and corona radiata region | < 0.0001*** |
| AD of corpus callosum region | 0.0031** |

Abbreviations: AD, axial diffusivity; FA, fractional anisotropy; PPT, persistent post-traumatic headache; ROI, region of interest.

*Significant at *P* ≤ 0.05. **Significant at *P* ≤ 0.01. ***Significant at *P* ≤ 0.001.

**Supplemental Figure 1. Significant Diffusion MRI Differences Between Persistent Post-Traumatic Headache and Migraine**


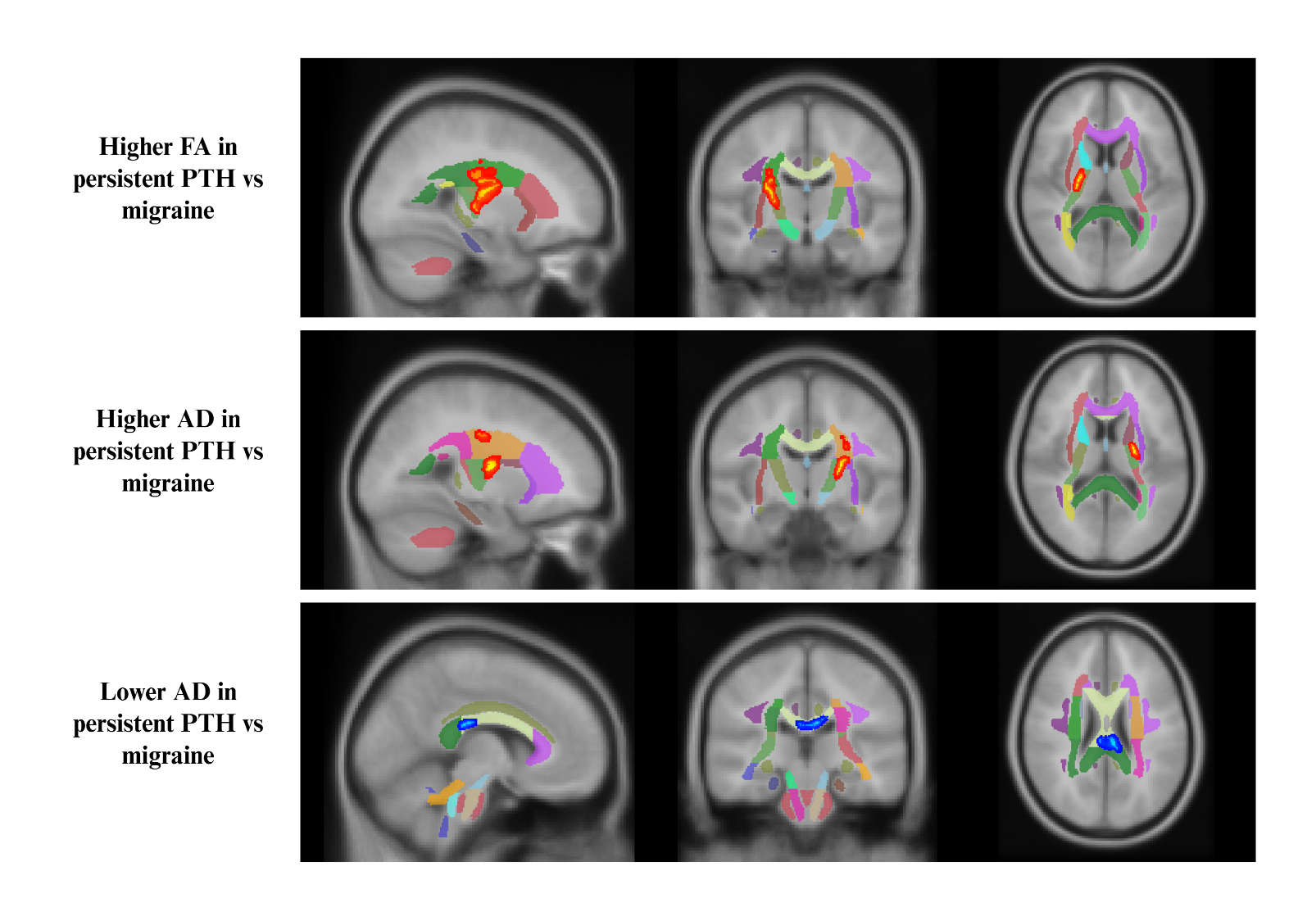


Significant voxels are overlaid on the Johns Hopkins University (JHU) International Consortium for Brain Mapping (ICBM) DTI-81 White-Matter Labels Atlas [3]. Upper panel: Higher fractional anisotropy (FA) in participants with persistent post-traumatic headache (PTH) compared to those with migraine (hot colors). Middle panel: Higher axial diffusivity (AD) in participants with PTH compared to migraine (hot colors). Lower panel: Lower FA in participants with PTH compared to migraine (cold colors). Significant voxels filled for display purposes.

**Supplemental Materials References**

1. Christensen RH, Al-Khazali HM, Ashina M, et al (2025) Differences in Cortical Morphometry between Persistent Post-Traumatic Headache, Migraine, and Healthy Controls. Cephalalgia (in press)

2. Christensen RH, Ashina H, Al-Khazali HM, et al (2024) Differences in Cortical Morphology in People With and Without Migraine: A Registry for Migraine (REFORM) MRI Study. Neurology 102:e209305. https://doi.org/10.1212/WNL.0000000000209305

3. Mori S, Oishi K, Jiang H, et al (2008) Stereotaxic White Matter Atlas Based on Diffusion Tensor Imaging in an ICBM Template. Neuroimage 40:570. https://doi.org/10.1016/J.NEUROIMAGE.2007.12.035
